# Supplementary material for: Predicting visual function by interpreting a neuronal wiring diagram
Source: Nature. 2024 Oct 2;634(8032):113–23. doi: 10.1038/s41586-024-07953-5 (PMC11446822; doi:10.1038/s41586-024-07953-5)
Supplement: Supplementary file 6 — CRF and ERF predictions for individual TmY4 and TmY9 cells. Analogous to Supplementary Data 3, but for TmY target types. Shown are the top four monosynaptic pathways, the strongest pathway passing through each of the top ten intermediary types (ranking from Extended Data Fig. 7), and the trisynaptic pathway Tm1–TmY–Dm3–TmY (see the section entitled Prediction of spatial normalization). [file 41586_2024_7953_MOESM6_ESM.zip › DataS4/TmY9qΓèÑ/720575940629424171.pdf]

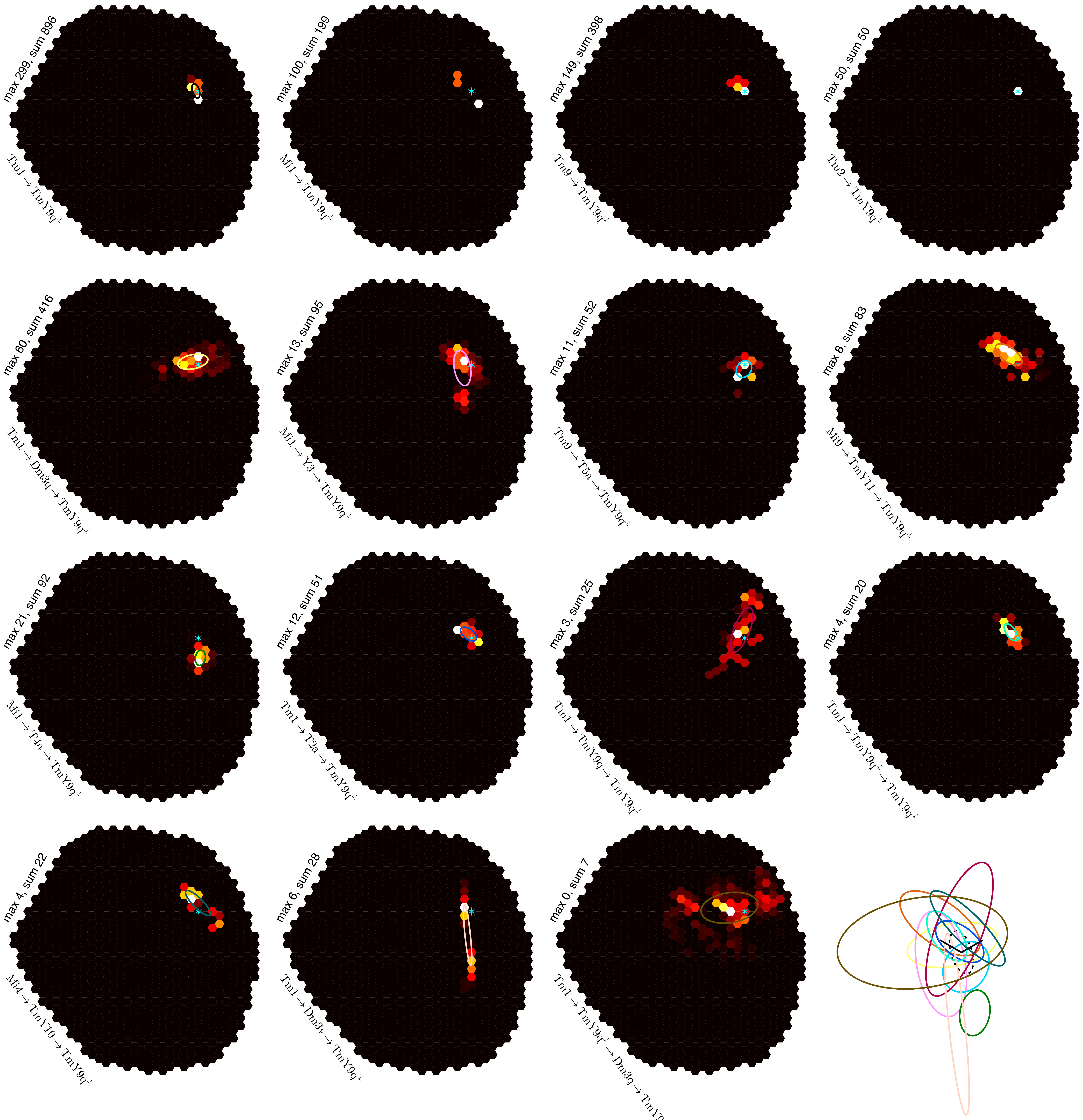
max 299, sum 896
Tm1 → TmY9q⊥
max 100, sum 199
Mi1 → TmY9q⊥
max 149, sum 398
Tm9 → TmY9q⊥
max 50, sum 50
Tm2 → TmY9q⊥
max 60, sum 416
Tm1 → Dm3q → TmY9q⊥
max 13, sum 95
Mi1 → Y3 → TmY9q⊥
max 11, sum 52
Tm9 → T5a → TmY9q⊥
max 8, sum 83
Mi9 → TmY11 → TmY9q⊥
max 21, sum 92
Mi1 → T4a → TmY9q⊥
max 12, sum 51
Tm1 → T2a → TmY9q⊥
max 3, sum 25
Tm1 → TmY9q → TmY9q⊥
max 4, sum 20
Tm1 → TmY9q⊥ → TmY9q⊥
max 4, sum 22
Mi4 → TmY10 → TmY9q⊥
max 6, sum 28
Tm1 → Dm3v → TmY9q⊥
max 0, sum 7
Tm1 → TmY9q⊥ → Dm3q → TmY9q⊥
